# 16SPIP: a comprehensive analysis pipeline for rapid pathogen detection in clinical samples based on 16S metagenomic sequencing

**DOI:** 10.1186/s12859-017-1975-3

**Published:** 2017-12-28

**Authors:** Jiaojiao Miao, Na Han, Yujun Qiang, Tingting Zhang, Xiuwen Li, Wen Zhang

**Affiliations:** 1 State Key Laboratory for Infectious Disease Prevention and Control, National Institute for Communicable Disease Control and Prevention, Chinese Center for Disease Control and Prevention, Beijing, 102206 China; 2 Collaborative Innovation Center for Diagnosis and Treatment of Infectious Diseases, Hangzhou, 310003 China

**Keywords:** 16S, High-throughput sequencing, Pathogens, 16SPIP, Comprehensive analysis pipeline

## Abstract

**Background:**

Pathogen detection in clinical samples based on 16S metagenomic sequencing technology in microbiology laboratories is an important strategy for clinical diagnosis, public health surveillance, and investigations of outbreaks. However, the implementation of the technology is limited by its accuracy and the time required for bioinformatics analysis. Therefore, a simple, standardized, and rapid analysis pipeline from the receipt of clinical samples to the generation of a test report is needed to increase the use of metagenomic analyses in clinical settings.

**Results:**

We developed a comprehensive bioinformatics analysis pipeline for the identification of pathogens in clinical samples based on 16S metagenomic sequencing data, named 16SPIP. This pipeline offers two analysis modes (fast and sensitive mode) for the rapid conversion of clinical 16S metagenomic data to test reports for pathogen detection. The pipeline includes tools for data conversion, quality control, merging of paired-end reads, alignment, and pathogen identification. We validated the feasibility and accuracy of the pipeline using a combination of culture and whole-genome shotgun (WGS) metagenomic analyses.

**Conclusions:**

16SPIP may be effective for the analysis of 16S metagenomic sequencing data for real-time, rapid, and unbiased pathogen detection in clinical samples.

## Background

Several recent public health emergencies caused by bacterial pathogens have caused public concern, e.g., a *Streptococcus suis* outbreak in Sichuan Province in 2006, *Salmonella* infections in the United States in 2009, a 2010 Haitian cholera outbreak, and outbreaks of enterohemorrhagic *Escherichia coli* in Germany (2011) and England (2016). Rapid pathogen screening is a key step for the effective control of infectious diseases as well as for the prevention of disease transmission and elimination of public panic. According to standard methods for bacterial identification in conventional microbiology laboratories, bacteria are first cultured in a suitable growth medium to obtain a pure culture and are then identified based on phenotypes or biochemical properties [1]. However, this method has limitations, such as the ability to detect only culturable microorganisms, long detection time, and high rates of false-positive and false-negative results [2]. Molecular diagnostic methods, such as polymerase chain reaction (PCR) and real-time PCR (RT-PCR), have reduced the turnaround time from the receipt of samples to the generation of final results. However, these methods require prior knowledge of pathogenic species that could be present in a sample and are not suitable for high-throughput screening [3]. 16S rRNA/rDNA metagenomic sequencing provides a new tool for the identification of pathogens, including those that are non-culturable or cannot be identified based on phenotypes. In comparison with conventional culture methods, 16S sequence analyses have a higher sensitivity for the detection of specific bacteria [4, 5] and accordingly can be used for accurate bacterial species identification [6].

Several important bioinformatics tools for 16S data analysis have been released, for example, QIIME, Mothur, SILVAngs, MEGAN, and AmpliconNoise [7]. Despite having the vast availability of algorithms, QIIME is widely used in microbial community diversity analysis [8], as well as MEGAN [9] and Mothur [10]. SILVAngs [11] is a web page analysis tool that based on the SILVA database for the OTU species classification, only supports the maximum 500 M sequencing data. And AmpliconNoise [12] is mainly used for 454 sequencing data. All these tools are useful and has been used in so many research projects. However, all these tools are focusing on taxonomy assignment and diversity research. Currently, a standardized comprehensive analysis pipeline from the receipt of clinical samples to the generation of the test report is lacking for use by clinicians.

In this project, we developed 16SPIP (16S Pathogenic Identification Process), a bioinformatics analysis pipeline that identifies pathogens in clinical samples based on 16S metagenomic sequencing results. To evaluate its utility for emergency situations, the pipeline was applied to different clinical samples for pathogen detection, and the pathogens were successfully identified to the species level.

## Methods

**Fig. 1:**
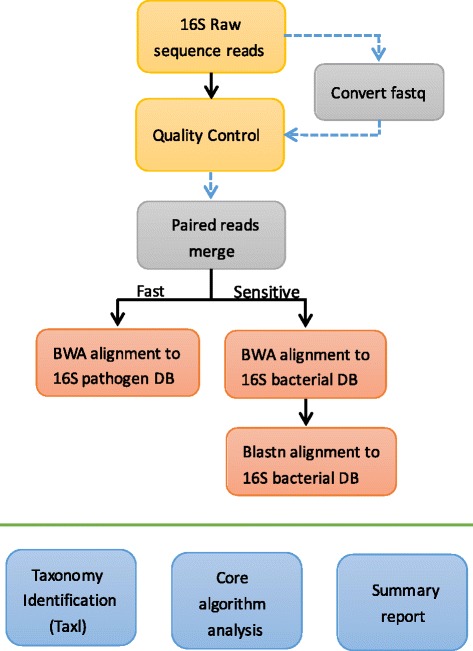
Flow chart of the 16SPIP analysis pipeline. During the pre-processing stage, 16S metagenomic raw data can either be used directly or subjected to file format conversion. The data were subjected to the removal of adapter, low-quality, and low-complexity sequences. The paired-end reads can either be merged using PEAR or used directly. In fast mode, pathogen identification was achieved by alignment against the 16S pathogen database using the BWA-MEM algorithm. In sensitive mode, the BWA-MEM algorithm was used for alignment against the 16S full-length database. Sequence reads with greater than 99% similarity to the reference sequence were extracted and re-aligned against the 16S full-length database using BLASTN. The test report generated from 16SPIP includes the basic information for the sample, pathogen detection results, and distribution of bacterial species

16SPIP is an integrative application that consists of a series of Perl, Python, and shell scripts, as well as next-generation sequencing (NGS) tools, including NGS QC Toolkit, Paired-End reAd mergeR (PEAR), Burrows-Wheeler Aligner (BWA), nucleotide BLAST (BLASTN), and Picard. 16SPIP accepts sequencing data (zipped or unzipped) from 454, Illumina and Ion torrent platforms in various formats, such as FASTQ, FASTA, SFF, SAM, and BAM, and data from multiple samples can be run in parallel to support rapid analysis. Figure 1 provides an overview of 16SPIP workflow. 16SPIP also provides complete flexibility to define all parameters and appropriate guidance to perform the analysis.

### 16S full-length reference database

All sequences with confirmed taxonomic relationships and read lengths ranging from 1200 bp to 2000 bp were selected from public 16S rDNA databases, such as Ribosomal Database Project (RDP) [13] and National Center for Biotechnology Information (NCBI), as well as a database constructed from full-length 16S rDNA fragments extracted from the complete genome sequences of nearly 100 pathogens that were collected, cultured, and sequenced by our department. After the removal of redundant sequences, the remaining 252,567 reads were used as the 16S full-length bacterial reference database in 16SPIP, covering 15,217 species and 2094 genera. This 16S bacterial database was used for alignment in the sensitive mode of 16SPIP.

### 16S pathogen database

16S pathogen database is a sub-database of the 16S full-length reference database. It was constructed from 29,258 total reads covering 346 bacterial species of human health concern in “The Directory of Pathogenic Microorganisms in Human Infections” published by the Ministry of Health of the People’s Republic of China. This 16S pathogen database was used for alignment in the fast mode of 16SPIP.

### Data import and quality control

16SPIP can determine the file format of raw NGS data. Picard [14] and the seq_crumbs [15] software package were integrated into the pipeline to convert NGS data from SAM, BAM, or SFF formats to the FASTQ format. Furthermore, quality scores from different sequencing platforms can be obtained during the pre-processing step. 16SPIP also accepts the FASTA format for raw NGS data. Read control procedures were based on the read length and ambiguous base (N) content. Quality control procedures included the removal of adapter sequences and trimming of low-quality bases. In order to obtain high-quality reads, the TrimmingReads.pl tool in the NGS QC Toolkit [16] software package was used to trim low-quality bases with PHRED scores of less than 20 (Q20) at the 3′ end of the read and to remove reads with lengths of less than 50 bp.

16SPIP can process NGS data obtained from both single-read and paired-end sequencing. For paired-end sequencing data, R1 and R2 reads from raw NGS sequence were processed according to the above-mentioned methods. Two high-quality sequence files with overlapping paired-end reads were then merged using PEAR [17]. For NGS data generated from single-read sequencing, the merging step was skipped; data were directly subjected to downstream analysis. Users can also skip the quality control steps using self-defined parameters.

### 16SPIP fast mode for pathogen identification

16SPIP provides both fast and sensitive modes for analysis. In fast mode, the BWA-MEM algorithm [18] was used to align the sequence reads against the 16S pathogen reference database. Reads that were >99% identical to sequences in the reference database were then mapped to species based on the bacterial taxonomy information associated with the reference sequence and output as the final result.

### 16SPIP sensitive mode for pathogen identification

In order to avoid the misidentification of bacterial species owing to short sequence reads or those with high similarity, a two-step algorithm was used in the sensitive mode of 16SPIP during the bacterial identification process. In the first step, the BWA-MEM algorithm [18] was used to align the sequence reads against a reference database. Sequence similarity for each read that mapped to reference sequence was calculated, and reads that were >99% identical to the reference sequence were extracted. Based on the bacterial taxonomy information associated with the reference sequence, the content and distribution of the bacterial flora in samples can be obtained. For example, reads that were >99% identical to the reference sequence can be mapped to the species level, those >97% identical can be mapped to the genus level, and those >95% identical can be mapped to the family level. The reads extracted in the first step were then re-aligned against the reference database using BLASTN [19] (E-value from 10^−10^ to 10^−20^). The strain information for the reference sequence for each mapped read with greater than 97% similarity was analyzed. Based on the results obtained in the previous step, scores were assigned to samples that could contain pathogens. Higher scores indicate a higher probability that pathogens are present.

## Results

The operation of 16SPIP is illustrated in a practical example below. On November 8, 2016, a pus sample from a patient with a hand infection in a Beijing hospital was collected using a sterile cotton swab and sent to our laboratory for culture. A Qiagen kit (St. Louis, MO, USA, QIAamp UCP Pathogen Mini Kit) was used to extract DNA, and the V3-V4 regions of the 16S gene were amplified. The MiSeq platform was used to perform paired-end 250 bp (PE250) sequencing. A total of 1,948,892 raw reads were generated. Quality control procedures and paired reads merge after the remaining 1,928,129 reads for subsequent analysis. 1,928,016 reads can mapped to 16S reference genome for pathogen classification and identification.

The fast mode of 16SPIP (default parameters) was used to perform analysis based on the 16S metagenomic sequencing data. The processing time for the entire procedure, including data quality control, merging, alignment, statistical analysis, and the generation of the test report, was 2 h and 32 min. The final results indicated the detection of *Streptococcus agalactiae.* The operation time required for data analysis using the sensitive mode of 16SPIP was 6 h, and *Lactobacillus iners, S. agalactiae,* and *Lactobacillus jensenii* were detected. In addition, 16S gene amplification and full-length sequencing of positive cultures in the laboratory confirmed the presence of *S. agalactiae* on the third working day and *L. jensenii* on the sixth working day.

In a comparison among the 16SPIP fast mode, 16SPIP sensitive mode, and laboratory cultures, *S. agalactiae* was positively identified using all three methods. Previous studies have reported that *S. agalactiae* is related to human health; therefore, it was confirmed as the pathogen-causing infection in this case.

In order to validate the reliability of the 16SPIP test results, WGS metagenomic sequencing was conducted using the same sample. The genomic sequence of *S. agalactiae* represented 6.825% of the 13.4 Gb of sequencing data. These data also supported the test results generated from the fast mode of 16SPIP, where the bacterial taxon was positively detected in the sample.

**Fig. 2:**
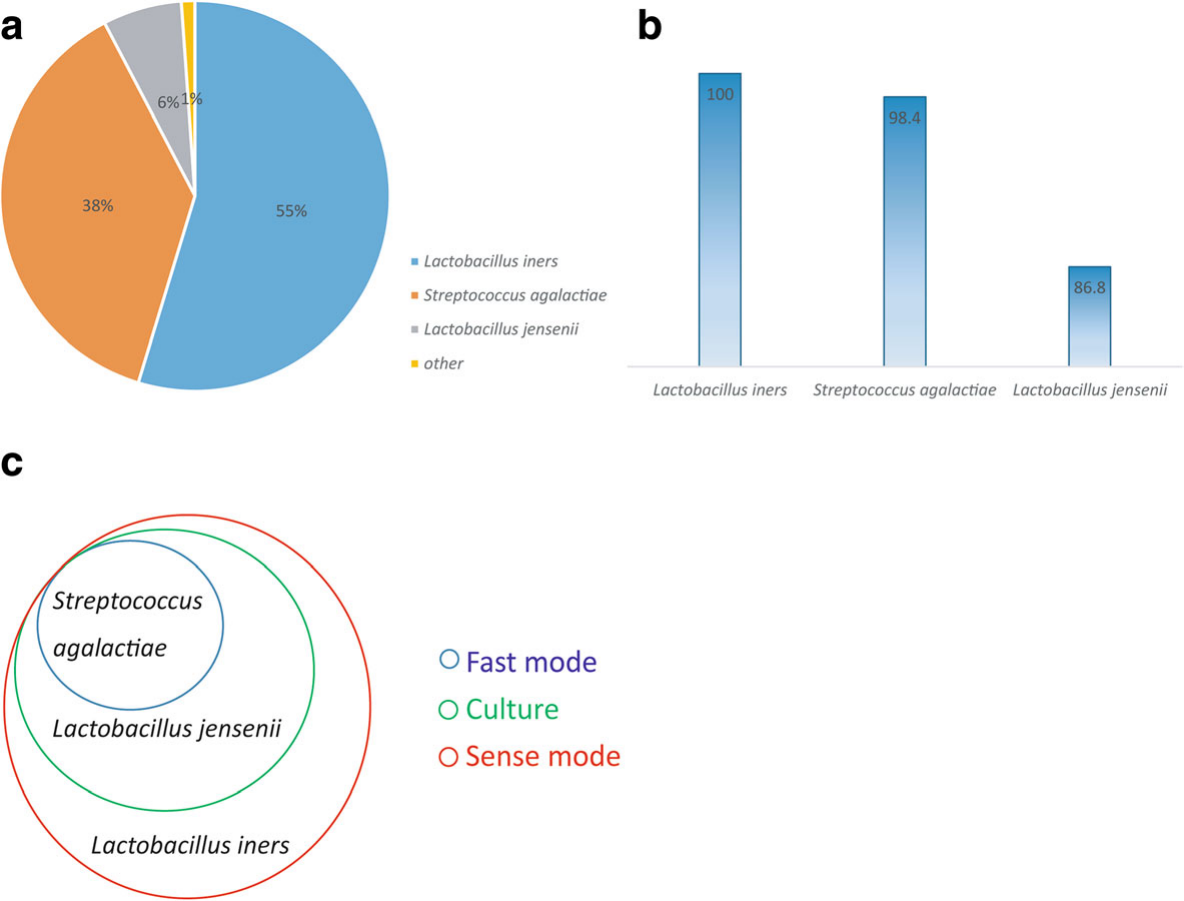
Results of pathogen detection for the pus swab. **a** Distribution of bacterial species in the sample. **b** Scores of pathogen detection from the analysis using the sensitive mode of 16SPIP. **c** Relationship between the results obtained using 16SPIP fast mode, 16SPIP sensitive mode, and bacterial culture

The sensitive mode of 16SPIP also detected the presence of *L. iners* and *L. jensenii. L. jensenii* was confirmed in the laboratory culture results. The missed detection of *L. iners* can probably be attributed to false-negative results in laboratory cultures. From another perspective, these results demonstrated the comprehensiveness of the 16SPIP test results. Figure 2 summarizes the results of pathogen detection for the pus swab example.

## Discussion

Owing to recent advances in NGS technology and the reduced cost of sequencing, 16S metagenomic sequencing has emerged as one of the most promising strategies for the detection of pathogens in clinical samples. However, the lack of professional bioinformatics analysts in most laboratories has limited the extensive use of 16S metagenomic sequencing for pathogen identification in clinical settings. Our goal was to establish a one-click analysis pipeline for pathogen identification in complex samples using 16S metagenomic data to provide a rapid and accurate tool for clinicians and the Centers for Disease Control and Prevention (CDC) staff.

## Conclusions

16SPIP is a comprehensive analysis pipeline with multiple integrated parts for the conversion of the data format, quality control, sequence filtering, rapid alignment, generation of the report, and other processes. It was designed with two analysis modes (i.e., the fast and sensitive mode) to enable usage under different working environments. In emergency situations, fast mode can be prioritized and combined with clinical symptoms to quickly screen for 346 pathogens associated with human health. If users want to identity the existence of other species as well as to study the population diversity of microbiome, the sensitive mode is the better choice.

With the support of the China CDC database, the accuracy of the analysis pipeline was validated in multiple practical cases. The source code of 16SPIP has been released on Github (https://github.com/jjmiao1314/16sPIP.git). Besides the local version, the web version of 16SPIP is available for academic users (http://16spip.mypathogen.cn/).

## Abbreviations

16SPIP: 16S Pathogenic Identification Process
BLASTN: Nucleotide BLAST
BWA: Burrows-Wheeler Aligner
*L. iners*: *Lactobacillus iners*
*L. jensenii*: *Lactobacillus jensenii*
N: Ambiguous base
NCBI: National Center for Biotechnology Information
NGS: Next-generation sequencing
PCR: Polymerase chain reaction
PE250: Paired-end 250 bp
PEAR: Paired-End reAd merger
rDNA: Ribosomal deoxyribonucleic acid
RDP: Ribosomal Database Project
rRNA: Ribosomal ribonucleic acid
RT-PCR: Real-time PCR
*S. agalactiae*: *Streptococcus agalactiae*
WGS: Whole-genome shotgun
